# New insight into the intravenous immunoglobulin treatment in Multisystem Inflammatory Syndrome in children and adults

**DOI:** 10.1186/s13052-024-01585-1

**Published:** 2024-01-25

**Authors:** Chih-Jen Chen, Hsu-Yen Kao, Ching-Hua Huang, Chia-Jung Li, Cheng-Hsien Hung, Su-Boon Yong

**Affiliations:** 1 Department of Pediatrics, Kaohsiung Chang Gung Memorial Hospital, Chang Gung University College of Medicine, Kaohsiung, Taiwan; 2 School of Medicine, Chung Shan Medical University, Taichung, Taiwan; 3 Institute of Medicine, Chung Shan Medical University, Taichung, Taiwan; 4 Department of Pharmacy, Chung Shan Medical University Hospital, Taichung, Taiwan; 5 Department of Obstetrics and Gynecology, Kaohsiung Veterans General Hospital, 813 Kaohsiung, Taiwan; 6 Institute of Biopharmaceutical Sciences, National Sun Yat-sen University, 804 Kaohsiung, Taiwan; 7 Department of Pharmacy, Chang Bing Show Chwan Memorial Hospital, 50544, No.6, Lugong Rd., Lukang Township, Changhua, Taiwan; 8 Department of Allergy and Immunology, China Medical University Children’s Hospital, No. 2, Yuh‑Der Road, 404 Taichung City, Taiwan

**Keywords:** IVIG, MIS-A, MIS-C

## Abstract

Within 6 months of the coronavirus pandemic, a new disease entity associated with a multisystem hyperinflammation syndrome as a result of a previous infection with the SARS-CoV-2 virus is increasingly being identified in children termed Multisystem Inflammatory Syndrome in Children (MIS-C) and more recently in adults(MIS-A). Due to its clinical similarity with Kawasaki Disease, some institutions have used intravenous immunoglobulins and steroids as first line agents in the management of the disease. We seek to find how effective intravenous immunoglobulin therapy is across these two disease entities. A comprehensive English literature search was conducted across PubMed, MEDLINE, and EMBASE databases using the keywords multisystem inflammatory syndrome in children/adults and treatment. All major online libraries concerning the diagnosis and treatment of MIS-C and MIS-A were searched. Relevant papers were read, reviewed, and analyzed. The use of intravenous immunoglobulins (IVIG) and steroids for the treatment of multisystemic inflammatory syndrome in children(MIS-C) is well established and recommended by multiple pediatric governing institutions. However, there is still no optimal treatment guideline or consensus on the use of IVIG in adults. The use of IVIG in both the child and adult populations may lower the risk of treatment failure and the need for adjunctive immunomodulatory therapy. Despite the promising results of IVIG use for the management of MIS-C and MIS-A, considering the pathophysiological differences between MIS-C and MIS-A, healthcare professionals need to further assess the differences in disease risk and treatment. The optimal dose, frequency, and duration of treatment are still unknown, more research is needed to establish treatment guidelines.

Severe acute respiratory syndrome coronavirus 2 (SARS-CoV-2), the virus responsible for severe acute respiratory syndrome, began its global spread in late 2019, and by April 2020, the first case of Multisystem Inflammatory Syndrome in Children (MIS-C) was reported [1]. Adults with similar symptoms and clinical manifestations were observed and eventually identified as Multisystem Inflammatory Syndrome in Adults (MIS-A) [2]. The cumulative cases of MIS-C in Taiwan have reached 129 patients as of September 2022, but not a case of MIS-A has been diagnosed that clearly meets the definition. Globally and in Taiwan, MIS-C appears to have a higher prevalence, estimated at 2 cases per 100,000 children, compared to MIS-A [3]. The incidence of MIS-C varies among different racial groups, with higher rates observed in African, African-Caribbean, and Hispanic patients, while lower rates are found in White and Asian populations [4, 5]. While our understanding of all the risk factors for MIS-C remains incomplete, we have identified several potential contributing factors, including race, age, obesity, and cancer [4, 6–8]. Moreover, Previous studies have shown that SARS-CoV vaccination is effective in reducing the incidence of MIS-C [9].

MIS-A is rarely reported [10]. Due to the lack of understanding of this syndrome and other inflammatory consequences of COVID-19, MIS-A may not be fully recognized. The relative rarity of MIS-A is consistent with the observation that MIS-C incidence decreases with age starting in adolescence. In one study, the occurrence of MIS-C among adolescents in the 16–20 age group was significantly less than that among children aged 5 years or younger [11]. In a retrospective study, it was determined that around 15 individuals, accounting for 9.6% of the 839 hospitalized patients fulfilled the criteria for MIS-A [12]. In another retrospective study, the CDC case definition [Fig. 1] was applied to search for MIS-A cases in electronic medical records. It was estimated that one in every 523 hospitalized COVID-19 patients had MIS-A. Out of the 11 identified patients, not a single one received intravenous immunoglobulin treatment [13]. The lack of recognition of MIS-A may result in missed opportunities for timely administration of medication.

**Fig. 1:**
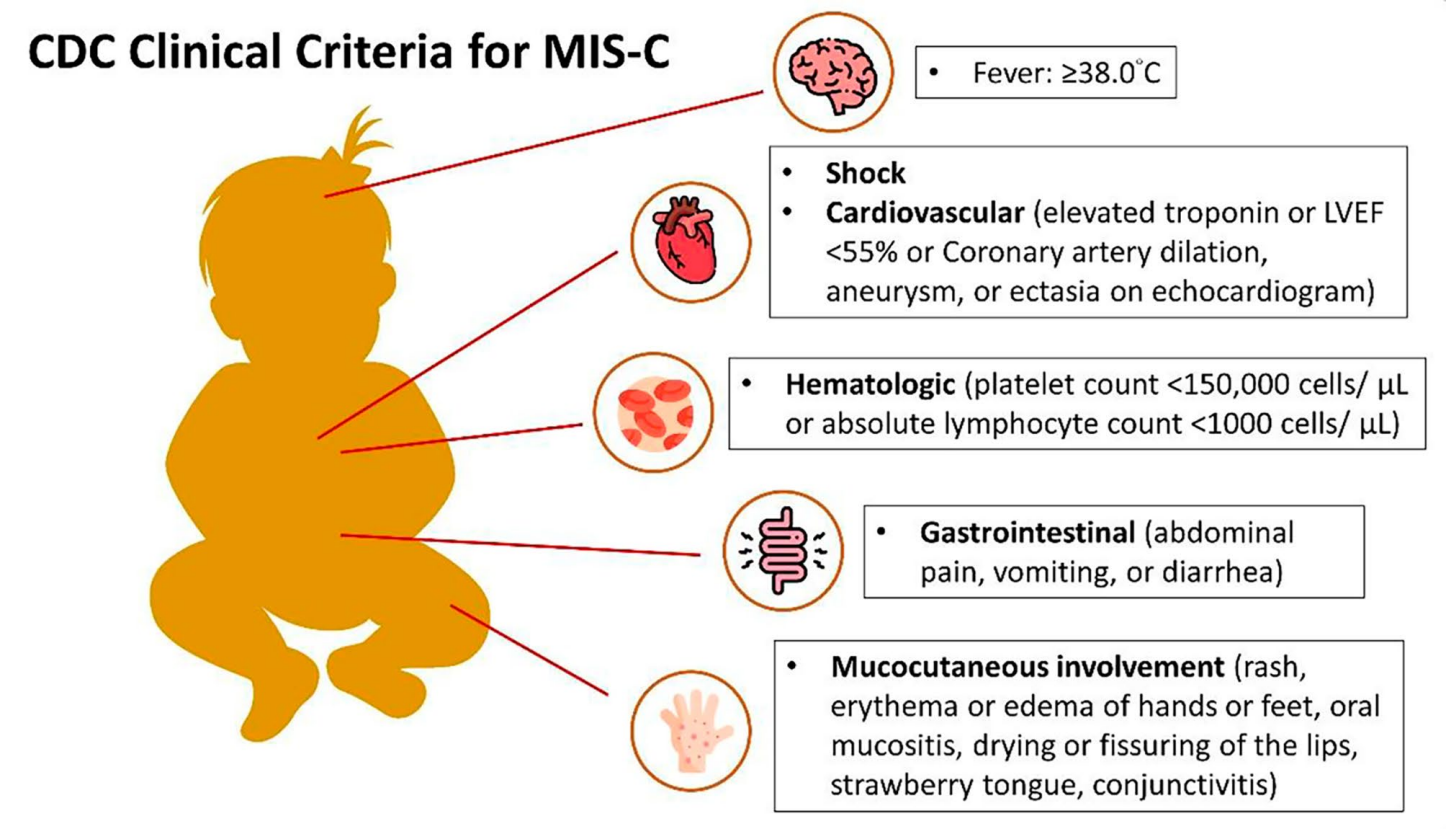
CDC Clinical Criteria for MIS-A The CDC Clinical Criteria for MIS-A are as follows: Subjective fever or documented fever (≥ 38.0 °C) for ≥ 24 h prior to hospitalization or within the first three days of hospitalization and at least three of the following clinical criteria occurring prior to hospitalization or within the first three days of hospitalization. At least one must be a primary clinical criterion. Primary clinical criteria include severe cardiac illness, which encompasses myocarditis, pericarditis, coronary artery dilatation/aneurysm, or new-onset right or left ventricular dysfunction (LVEF < 50%), 2nd/3rd degree A-V block, or ventricular tachycardia (Note: cardiac arrest alone does not meet this criterion), as well as the presence of rash AND non-purulent conjunctivitis. Secondary clinical criteria consist of new-onset neurologic signs and symptoms, such as encephalopathy in a patient without prior cognitive impairment, seizures, meningeal signs, or peripheral neuropathy, shock or hypotension not attributable to medical therapy, abdominal pain, vomiting, or diarrhea, and thrombocytopenia

Comparing MIS-C and MIS-A, some similarities and differences in diagnosis criteria, symptoms, and pathology were found. As outlined by the Centers for Disease Control (CDC), the criteria for MIS-C [Fig. 2] encompass individuals under the age of 21 who exhibit a fever exceeding 38.0 °C for a duration exceeding 24 h (with a median duration of four to six days), along with laboratory findings indicating inflammation and signs of severe clinical illness involving multiple organ systems (involving two or more systems, including cardiac, renal, respiratory, hematologic, gastrointestinal, dermatologic, or neurological) [3, 14]; no other credible alternative diagnoses, coupled with a positive confirmation of current or recent SARS-CoV-2 infection or exposure to COVID-19 within the four weeks preceding the onset of symptoms [1, 3, 15]. As for symptoms, the main symptoms of MIS-C include fever presenting ≥ 4 days, sixty to one hundred% having Gastrointestinal symptoms, bloodshot eyes, skin rash, diarrhea, etc., which is similar to Kawasaki disease (KD) [14, 15]. Many of the criteria for MIS-A resemble those of MIS-C, with the exception of age (where the individual should be over 21 years old). However, the diagnosis for MIS-A is more intricate than that for MIS-C, and the onset typically occurs approximately three weeks following diagnosis [2, 3]. The majority of individuals with MIS-A exhibit symptoms such as fever, low blood pressure, cardiac impairment, breathing difficulties, and/or diarrhea [2, 10].

**Fig. 2:**
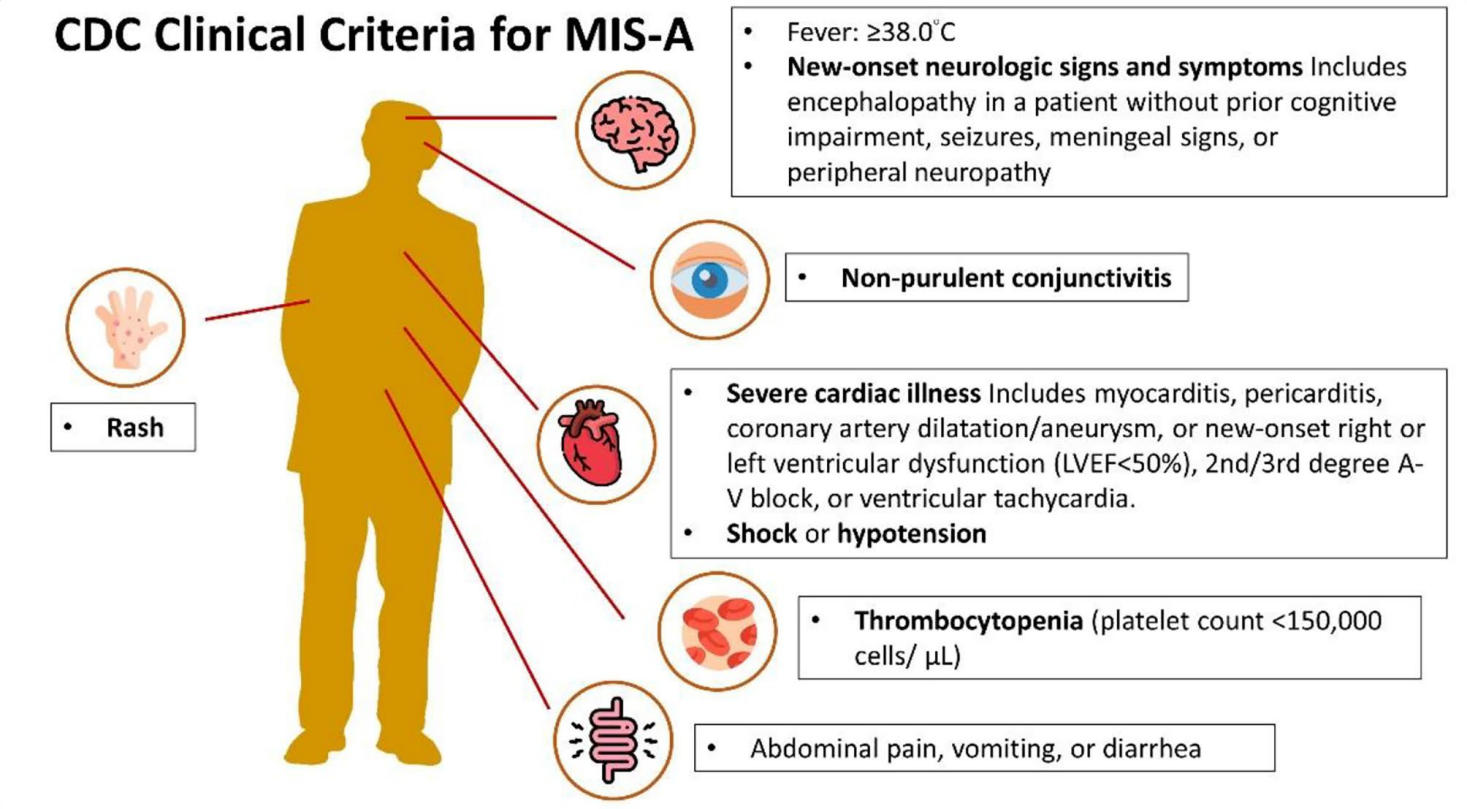
CDC Clinical Criteria for MIS-C The CDC Clinical Criteria for MIS-C are as follows: In the absence of a more likely alternative diagnosis, MIS-C is characterized by subjective or documented fever (temperature ≥ 38.0 °C), clinical severity requiring hospitalization or resulting in death, evidence of systemic inflammation indicated by C-reactive protein ≥ 3.0 mg/dL (30 mg/L), and new onset manifestations in at least two of the following categories. These categories include cardiac involvement, which can be identified by left ventricular ejection fraction < 55%, coronary artery dilatation, aneurysm, or ectasia, or elevated troponin levels above the laboratory’s normal range or indicated as elevated in a clinical note. Mucocutaneous involvement may manifest as a rash, inflammation of the oral mucosa, conjunctivitis, or conjunctival injection, as well as extremity findings like erythema or edema of the hands or feet. Shock is also considered. Gastrointestinal involvement is indicated by symptoms such as abdominal pain, vomiting, or diarrhea. Hematologic involvement is characterized by a platelet count < 150,000 cells/µL or an absolute lymphocyte count (ALC) < 1,000 cells/µL

MIS-C is thought to result from a post-inflammatory reaction following SARS-CoV-2 infection, showing notable similarities with other conditions like Kawasaki disease, toxic shock syndrome, and myocarditis [16]. This is likely due to the activation and dysregulation of common inflammatory pathways, which can result in clinical disease despite different underlying causes [17]. There are many similarities between MIS-C and KD. According to ACR guidelines, the incidence of MIS-C is increased in African, Afro-Caribbean, and Hispanic patients, while it is lower in East Asian patients compared to Kawasaki disease (KD) patients. The age range of MIS-C patients is wider than that of KD patients. When seeking medical care, MIS-C patients often exhibit platelet counts, absolute lymphocyte counts, and CRP levels that are typically lower than those observed in KD patients [18]. Ventricular dysfunction is a more prevalent finding in MIS-C, whereas KD tends to manifest more frequently with coronary artery aneurysms [19]. Based on a recent systematic review of the literature, MIS-C has distinct characteristics when compared to KD, such as more prevalent respiratory and gastrointestinal involvement, shock, increased incidence of cardiac complications, reduced incidence of conjunctival inflammatory symptoms, and elevated levels of inflammatory markers, myocardial injury markers, and creatine. Given the ongoing COVID-19 pandemic, clinicians should maintain a high degree of suspicion for this severe form of KD [20].

In the case of MIS-A, there were higher instances of severe cardiac dysfunction, potential arterial or venous thrombosis, and increased mortality [3, 21]. The actual mechanism of MIS-C/A still needs more research, but there are a few hypotheses. Current research suggests that the pathogenesis of MIS-C involves early SARS-CoV-2 infections in children, whether asymptomatic or with mild symptoms, potentially triggering macrophage activation and the stimulation of helper T cells. This, in turn, leads to the release of cytokines or a cytokine storm, further stimulating macrophages, neutrophils, and monocytes. Additionally, it activates B-cells and plasma cells, resulting in antibody production, ultimately culminating in a hyperimmune response [1, 16]. As a result, MIS-C is regarded as a delayed immunological event linked to inflammation [1]. MIS-A contributes to similar pathogenesis, although the balance between antiviral and proinflammatory responses in adults may be negatively influenced by age, thus leading to hyperinflammation [21].

Newborns born to mothers who were infected with SARS-CoV-2 during pregnancy have recently exhibited signs of a condition called multisystem inflammatory syndrome of the newborn (MIS-N). This condition is characterized by increased levels of inflammatory markers and affects multiple organs, with a particular emphasis on cardiac dysfunction [22]. In MIS-N cases, most neonates experience involvement of multiple systems, have elevated inflammatory markers, and test positive for IgG-SARS-CoV-2. However, it is important to note that a lack of elevated IgM cannot reliably rule out acute infection in newborns, particularly in premature infants who may have an underdeveloped immune system and may not be capable of producing an IgM response [23]. The diagnostic criteria for Multisystem Inflammatory Syndrome in neonates (MIS-N) are still evolving and subject to controversy. Additionally, due to the potential risk of necrotizing enterocolitis, caution is advised during intravenous immunoglobulin (IVIG) treatment in neonates [24].

Most drugs currently available for treating MIS are reported as case reports or case series. No randomized controlled trials have been identified. The main drugs studied include immunoglobulins, glucocorticoids, monoclonal antibodies, anticoagulants, and antiplatelet agents [25]. Due to the lack of data from randomized controlled trials, international organizations such as the WHO and the American College of Rheumatology have developed treatment guidelines to aid in the prompt diagnosis and treatment of children with MIS-C symptoms. Typically, these guidelines suggest initiating intravenous immunoglobulin (IVIG) therapy as the primary treatment for MIS-C, drawing on the experience gained from IVIG usage in Kawasaki disease [26]. According to the recommendations of the American College of Rheumatology (ACR) Clinical Guidance, drug therapy for MIS-C includes the use of intravenous immunoglobulin (IVIG) and steroids. If patients require additional treatment, they may opt for biologics or other immunomodulatory therapies such as anakinra, tocilizumab, or baricitinib [19]. The primary treatment approach for patients with MIS-C involves the use of immunomodulatory medications, including intravenous immunoglobulin (IVIG), which is a blood product containing antibodies from numerous healthy donors, as well as corticosteroids [2, 27, 28]. These antibodies in IVIG can help to calm the immune system and reduce inflammation. IVIG has been used for decades to treat a variety of immune-mediated conditions, including Kawasaki disease, which is believed to be similar to MIS-C. In line with consensus guidelines, MIS-C patients typically receive intravenous immunoglobulin (IVIG) at a dosage of 2 g/kg, calculated based on ideal body weight, with a maximum limit of 100 g [28, 29]. In addition, for critically ill COVID-19 patients, a short-term, low-dose glucocorticoid regimen is typically recommended, with methylprednisolone administered at 1–2 mg/kg/day over a period of 3 to 5 days [28, 30]. Medication treatment for MIS-C is in Table 1.

**Table 1:** Medication treatment for MIS-C

Medications	Management	Drugs	Usual dosage
Immunoglobulins	Immunomodulatory: IVIG considered first-line therapy.	IVIG [19, 51]	Single dose at 2 gm/kg based on ideal body weight
Glucocorticoids	Immunomodulatory: Glucocorticoids should be used as adjunctive therapy with severe disease or refractory disease.	Methylprednisolone [19, 52]	1–2 mg/kg/day, typically in two doses
		Dexamethasone [19, 53]	0.15–0.4 mg/kg/day, PO or IV
		Prednisone [28]	1–2 mg/kg/day, PO
Monoclonal antibodies	Immunomodulatory: Lack of response to the treatment.	Anakinra [19, 29]	Starting dosing at 2–3 mg/kg q12h SC (total of 4–6 mg/kg/day, max: 100 mg/dose)
		Tocilizumab [19]	Single IV dose (< 30 kg: 12 mg/kg IV; ≥ 30 kg: 8 mg/kg IV; max: 800 mg)
		Infliximab [19, 54]	5–10 mg/kg, 1 dose
Anticoagulants	Anticoagulant thromboprophylaxis	Enoxaparin [19, 55]	1 mg/kg/day
		Warfarin [19]	Therapeutic anticoagulation with warfarin
Antiplatelet agents	Antiplatelet therapy	Low-dose aspirin [19, 56]	3–5 mg/kg/day, max: 81 mg/day

IVIG: Immunoglobulins; LMWH: Low-Molecular-Weight Heparin

Although some cases that were treated with corticosteroids and therapeutic anticoagulation successfully recovered [2], there is still no optimal treatment strategy for MIS-A to date. Many anti-inflammatory treatments are currently used [31]. There is insufficient evidence that the combination of IVIG and corticosteroids is beneficial for MIS-A patients [32]. There are currently no consensus treatment guidelines for MIS-A, however a recent review article with a sample size of 79 MIS-A patients, reported on the use of steroids (60.2%), intravenous immunoglobulin (37.2%), and biologics (10.2%) with an overall mortality rate of 5.1% [10]. From current evidence, unrecognized MIS-A has a high mortality rate. The prognosis of this disease depends on early recognition of the condition and rapid implementation of immunomodulatory therapy (steroids, immunoglobulins) [33]. Early management reduces the risk of serious and life-threatening complications. The current treatment strategy for MIS-A is derived from the treatment protocols used for MIS-C [34]. Steroids and IVIG are considered effective first-line therapies, with further consideration of other immunomodulatory drugs for patients with refractory MIS-A.

In contrast to corticosteroids, which are a class of non-inflammatory steroidal hormones, IVIG is a collective product of normal IgG immunoglobulin gathered from thousands of healthy donors [30]. The precise mechanism of IVIG in treating MIS-C/MIS-A is still the subject of ongoing research. Nonetheless, some proposed mechanisms involve the neutralization of pathogenic autoantibodies that interact with macrophage Fc receptors and the inhibition of autoantibody binding to macrophages [35]. Besides, IVIG can also induce anti-inflammatory cytokines and inhibit the activation and proliferation of B cells [30], which is important for suppressing inflammation. Moreover, corticosteroids are used in severe COVID-19 cases with cytokine storm. The timing and dosage of corticosteroid administration is very crucial, as it may increase the viral load, and eventually connive adverse effects [30]. Choudhary et al. reported that high-dose IVIG (2 g/kg) was not indicated in severe COVID-19 patients or other coronaviruses [30], while a cohort study by Wang et al. suggested that short-term and low-dose methylprednisolone is better for severe COVID-19 patients than high dose corticosteroids [36].

Nonetheless, studies suggested using combination therapy [10, 37]. In a one-year follow-up of MIS-C patients, more (57%) were recovered from both IVIG + methylprednisolone (combination) treatment, compared to the ones recovered from only IVIG (1.8%) and only methylprednisolone (18%) [38]. An observational study in Singapore emphasized that early initiation of treatment with IVIG and steroids likely contributed to comparatively good outcomes [39]. In a study published in the New England Journal of Medicine, initial treatment with IVIG plus glucocorticoids was also associated with a lower risk of new or persistent cardiovascular dysfunction than IVIG alone [31]. Even the consensus guidelines from the American College of Rheumatology (ACR) for MIS-C advise considering the use of low-to-moderate dose intravenous corticosteroids (typically methylprednisolone at 1–2 mg/kg/day) in conjunction with IVIG when managing cases of shock or organ-threatening disease [28]. These researches show that the use of the combination of IVIG and corticosteroid has benefits, including shorter recovery time, a lower risk of cardiovascular dysfunction, and a significantly decreased risk in the use of immunomodulatory treatment [28, 40, 41]. Combination therapy may be a better treatment strategy for patients with MIS-C, however, MIS-A requires more diverse therapies. In a recent large cohort study, the effectiveness of different treatments for 2101 children diagnosed with MIS-C was compared. The authors applied two different propensity score methods to address potential bias due to differences in severity, demographics, or resource settings. The recovery rates, including the occurrence and regression of coronary artery aneurysms, were found to be similar for the primary treatment with intravenous immunoglobulin, compared with glucocorticoids or a combination of both. Given the cost and availability of intravenous immunoglobulin, initial treatment with glucocorticoids appears to be a safe alternative to immunoglobulin or combination therapy [26].

Although information on the use of IVIG for MIS-A is still limited, positive treatment outcomes have been reported based on case reports [42–46]. Both therapies alleviate inflammation, but the dosage of IVIG and corticosteroid should be determined. A recent study done in the United Kingdom using the Delphi method reported that, in MIS-A cases where there is evidence of coronary artery abnormalities or toxic shock syndrome, the IVIG dose should be 2 g/kg, calculated based on ideal body mass index. The administration can be in a single or divided dose, depending on the clinical presentation and cardiac function [47]. The course and symptoms of COVID-19 disease in children and adults are not completely similar, and the pathophysiology of MIS-C and MIS-A remains largely unknown. More evidence is needed to support the efficacy of IVIG in treating MIS-A.

Even though numerous clinical trials have demonstrated the efficacy and tolerability of immunoglobulin, various adverse effects have been reported. These include transient and mild symptoms such as flushing, headache, discomfort, fever, chills, fatigue, and drowsiness. However, rare and serious adverse reactions have also been reported, such as renal impairment, thrombosis, and hemolytic anemia, which are associated with specific immunoglobulin preparations and individual differences. Therefore, it is the clinician’s responsibility to determine individualized doses to ensure therapeutic efficacy and minimize adverse effects [48]. A previous systematic review of literature on the use of IVIG therapy in hospitalized adult patients with COVID-19 found no significant benefits on patient mortality or length of stay. Furthermore, prospective studies suggest that IVIG therapy may increase the length of stay in critically ill COVID-19 patients. Therefore, the accurate identification of adult patients who require IVIG therapy is a clinically important issue [49].

## Conclusion

In conclusion, the current knowledge of MIS-C is still ongoing. IVIG is effective for MIS-C, and the adjuncts might also serve as an effective treatment strategy for MIS-A. Despite the promising results, many questions remain about the best way to use IVIG in MIS-C and MIS-A. The optimal dose, frequency, and duration of treatment are not yet known. Additionally, there is a shortage of IVIG and it is expensive. This has led to some hospitals rationing the treatment and only administering it to the most severe cases. Moreover, given the limited available data on MIS-A, there might be a need to reference the treatment and pathophysiological model used for MIS-C when managing adult patients [10]. IVIG might be a promising treatment option for children and adults with MIS-C and MIS-A, but more research is needed to determine the best way to use it. The disease risk needs to be tracked more closely, and large-scale studies are needed to establish treatment guidelines [50]. In the meantime, efforts should be made to increase the availability of IVIG and to make it more affordable for patients in need. At the same time, it is important to continue to focus on preventing COVID-19 infection through vaccination and other measures to reduce the number of cases of MIS-C and MIS-A.

## Data Availability

Not applicable.

## Abbreviations

IVIG: intravenous immunoglobulins
MIS-C: Multisystem Inflammatory Syndrome in Children
MIS-A: Multisystem Inflammatory Syndrome in Adult
KD: Kawasaki disease
MIS-N: Multisystem Inflammatory Syndrome of the Newborn
